# Extracellular Vesicles isolated from Mesenchymal Stromal Cells Modulate CD4^+^ T Lymphocytes Toward a Regulatory Profile

**DOI:** 10.3390/cells9041059

**Published:** 2020-04-23

**Authors:** Flavia Franco da Cunha, Vinicius Andrade-Oliveira, Danilo Candido de Almeida, Tamiris Borges da Silva, Cristiane Naffah de Souza Breda, Mario Costa Cruz, Eliana L. Faquim-Mauro, Marcos Antonio Cenedeze, Meire Ioshie Hiyane, Alvaro Pacheco-Silva, Regiane Aparecida Cavinato, Ana Claudia Torrecilhas, Niels Olsen Saraiva Câmara

**Affiliations:** 1 Departamento de Nefrologia, UNIFESP, Rua Pedro de Toledo 669, São Paulo 04039-032, Brazil; gudaalmeida@gmail.com (D.C.d.A.); tammibscot@gmail.com (T.B.d.S.); mcenedeze@gmail.com (M.A.C.); apachecosf@gmail.com (A.P.-S.); regiane.cavinato@gmail.com (R.A.C.); 2 Departamento de Imunologia, USP, Avenida Prof. Lineu Prestes 1730, ICB IV, São Paulo 05508-000, Brazil; andradevinicius1@gmail.com (V.A.-O.); cris.naffah@gmail.com (C.N.d.S.B.); costacruzmc@gmail.com (M.C.C.); miy@icb.usp.br (M.I.H.); 3 Laboratório de Imunopatologia, Instituto Butantan, Av. Vital Brasil 1500, São Paulo 05503-900, Brazil; eliana.faquim@butantan.gov.br; 4 Hospital Israelita Albert Einstein, Av. Albert Einstein, São Paulo 627–05652-900, Brazil; 5 Departamento de Ciências Farmacêuticas, UNIFESP, Rua São Nicolau 210, Diadema 09913-030, São Paulo, Brazil; ana.torrecilhas@unifesp.br

**Keywords:** mesenchymal stromal cells, extracellular vesicles, Th1 polarization, miRNA, metabolism

## Abstract

Mesenchymal stromal cells (MSCs) can generate immunological tolerance due to their regulatory activity in many immune cells. Extracellular vesicles (EVs) release is a pivotal mechanism by which MSCs exert their actions. In this study, we evaluate whether mesenchymal stromal cell extracellular vesicles (MSC-EVs) can modulate T cell response. MSCs were expanded and EVs were obtained by differential ultracentrifugation of the supernatant. The incorporation of MSC-EVs by T cells was detected by confocal microscopy. Expression of surface markers was detected by flow cytometry or CytoFLEX and cytokines were detected by RT-PCR, FACS and confocal microscopy and a miRNA PCR array was performed. We demonstrated that MSC-EVs were incorporated by lymphocytes in vitro and decreased T cell proliferation and Th1 differentiation. Interestingly, in Th1 polarization, MSC-EVs increased Foxp3 expression and generated a subpopulation of IFN-γ^+^/Foxp3^+^T cells with suppressive capacity. A differential expression profile of miRNAs in MSC-EVs-treated Th1 cells was seen, and also a modulation of one of their target genes, *TGFbR2*. MSC-EVs altered the metabolism of Th1-differentiated T cells, suggesting the involvement of the TGF-β pathway in this metabolic modulation. The addition of MSC-EVs in vivo, in an OVA immunization model, generated cells Foxp3^+^. Thus, our findings suggest that MSC-EVs are able to specifically modulate activated T cells at an alternative regulatory profile by miRNAs and metabolism shifting.

## 1. Introduction

Mesenchymal stromal cells (MSCs) are adherent cells, capable of proliferating and differentiating in mature cells of mesenchymal lines [1] and expressing CD73, CD90, CD105 [2]. MSCs exert different biologic functions, which include, besides cell differentiation in multiple lines, tissue repair and immunosuppression. MSCs can modulate innate cells such as monocytes and macrophages, DCs and NK cells [3] and cells of the adaptive immune system, preventing the proliferation of CD4^+^ and CD8^+^ T cells and B cells. The effect of MSCs on T cells modulation is more widely studied. These cells suppress the proliferation of CD4^+^ and CD8^+^ naïve and memory T cells [4,5]. The presence of MSCs in lymphocyte culture may also lead to increase of regulatory T cell subpopulations (Treg) [6,7,8,9], a subtype essential for the suppression of immune response and tolerance induction [10]. Studies that pursue to identify the mechanisms by which MSCs exert their regulation suggest that the paracrine effect is more important than cell-cell contact, being the main mediator of this action [3]. In this context, the release of soluble factors with immunomodulatory properties, such as HGF [11], TGF-β [7], IL-10 [12], prostaglandin-E_2_ (PGE_2_) [13], indoleamine-2,3-dioxygenase (IDO) [14], has been identified as responsible for the effects of MSCs in several studies. Recently, nevertheless, the release of extracellular vesicles (EVs) by these cells has been demonstrated as an alternative mechanism by which MSCs perform their biologic effects [15].

EVs include several particles which are classified according to their origin and size. Exosomes are small particles (40 to 100 nm in diameter), derived from the endocytic pathway, released through the fusion of multivesicular bodies (MVBs) with the cell membrane [16]. Microvesicles (MVs) are larger particles (50 to 1000 nm in diameter) and more heterogeneous, originating from the direct budding of the plasma membrane [17]. Apoptotic bodies and oncosomes are vesicles of larger size (>1 μm). Apoptotic bodies are released after apoptotic cells fragmentation [18], while oncosomes are produced by the membrane protrusion of malignant cells [19]. More recently, a new subtype has been identified, the exomeres, with approximately 35 nm, these particles are enriched of proteins involved in cell metabolism [20]. EVs contain proteins, RNAs and miRNAs, DNAs and lipids that can be transferred to target cells. The composition of EVs may change according to tissue and cell type of origin, as well as their physiological status [21]. Once captured by the target cells, EVs can release their contents into the cytosol, being the transfer of active biomolecules the most responsible for their biologic effect. Hence, EVs are able to modify or reprogram the recipient cells.

In this sense, like the cells of origin, mesenchymal stromal cell extracellular vesicles (MSC-EVs) have been widely studied as a therapeutic option for different diseases. It was shown that MSC-EVs contains different mRNAs and miRNAs that can be transferred to other cells [22,23]. mRNAs involved in the control of transcription, proliferation and immunoregulation [22,23] have already been identified in these EVs, as well as miRNAs involved in the development of multiple organs, cell survival and differentiation. An important group of miRNAs associated with the regulation of the immune system was also found [24]. It was observed that MSC-EVs administration reduce inflammation, primarily by reducing infiltration of inflammatory cells as macrophages [25], leukocytes and neutrophils [26]. In a recent study, our group showed the capacity of MSC-EVs in modulate activated macrophages using a model of thioglycolate-induced peritonitis. The treatment with MSC-EVs decreased the macrophages infiltration and increased M2 polarization [27].

In this present study, we hypothesized that MSC-EVs are able to modulate immune cells, especially lymphocytes, leading to a regulatory profile and generating a condition of immunological tolerance that may be beneficial in cases of different inflammatory diseases. Therefore, we demonstrated that MSC-EVs were able to modulate lymphocytes proliferation and Th1 differentiation, leading to an alternative regulatory profile. This modulation was controlled by changing in miRNA profile and T cell metabolism, associated to the regulation of TGF-β pathway.

## 2. Materials and Methods

### 2.1. Animals

All animal experiments were carried out in the vivarium of Federal University of São Paulo (UNIFESP) in accordance with Federal Law 6638 of 1979, which regulates the use of animals in scientific experimentation, under approval of the Research Ethics Committee of the UNIFESP (CEUA 9031100214) and Instituto Butantan (CEUAIB 5954100918). Eight-to-nine-week C57BL/6 wild-type and Foxp3-GFP knock-in mice were obtained from the Center for the Development of Experimental Models for Medicine and Biology—CEDEME of the Federal University of São Paulo—UNIFESP being kept with light/dark artificial cycle of 12 h, at a constant temperature of 22 °C. Autoclaved water and food were supplied ad libitum.

### 2.2. Isolation and Characterization of MSC-EVs

MSCs were isolated from the adipose tissue of C57BL/6 mice and maintained in DMEM low-glucose, 10% fetal bovine serum (FBS) (Hyclone), 100-U/mL penicillin and streptomycin (Gibco). Cells were incubated at 37 °C in a humidified atmosphere with 5% CO_2_. Cultures with passages between 15 to 20 at confluence of 90% had its media substituted for DMEM low glucose without FBS and further, the supernatant was collected after 48 h. To obtain EVs, these supernatants were pre-centrifuged at 2000× *g* for 20 min at 4 °C, to exclude larger particles and cellular debris, and then ultracentrifuged at 100,000× *g* for 2 h at 4 °C. After ultracentrifugation, MSC-EVs were resuspended in PBS and stored at −80 °C. Characterization of EVs was done according to “Minimal Information for Studies of Extracellular Vesicles” (MISEV) [28,29]. To determine EVs concentration, MSC-EVs were diluted 500X and visualized and characterized for size, distribution and concentration using the Nanoparticles tracking analyses (NTA) (Malvern, UK) and Zetasizer (Malvern, UK) systems. MSC-EVs were labeled with surface molecules expressed by EVs (CD9—clone: KMC8—and annexin—BD catalog 51-65874X) and MSCs (CD45 clone: 30-F11, CD90 clone: G7, CD73 clone: TY-23, CD105 clone: MJ7/18) with specific antibodies and analyzed by flow cytometry and CytoFLEX (Beckman Coulter) and the CytExpert software (Beckman Coulter).

### 2.3. Scanning Electron Microscopy (SEM)

MSCs were plated in glass coverslips in 24 wells plate and after they reached 60% of confluence the cells were washed and added media without FBS. After 48 h, cells were fixed in a 2.5% glutaraldehyde solution as reported elsewhere [30]. The cells were post fixed with osmium tetroxide, treated with tannic acid, and dehydrated with ethanol. Samples were observed in a Field Emission FEI Quanta 250 FEG scanning electron microscope (FEI, OR, USA).

### 2.4. Transmission Electronic Microscopy (TEM)

After ultracentrifugation, MSC-EVs were resuspended in a 2% paraformaldehyde solution. The particles suspension was dripped onto carbon-coated electron microscopy screens and adsorbed for 20 min. The screens were fixed with glutaraldehyde 1% and washed with deionized water. Subsequently, the screens were contrasted with uranyl acetate for 10 min and rinsed again with distilled water and air dried. The images were acquired and observed in a JEOL 1200 EX II transmission electron microscope at 80 kV.

### 2.5. Detection and Incorporation of EVs

MSC-EVs were labeled with the fluorescent red dye PKH26 (Sigma) following the manufacturer’s instructions and subjected to ultracentrifugation for the washes required to remove excess of dye. Labeled EVs were added to the culture of naive CD4^+^ T lymphocytes purified by FACS sorting (FacsAria-BD) and activated with anti-CD3 (BD—clone145-2C11) and anti-CD28 (BD—clone 37.51) for evaluation of the internalization through the imaging by confocal microscopy (Zeiss LSM 780-NLO). Lymphocytes were monitored overnight for approximately 15 h.

### 2.6. T Cell Isolation and Total Splenocytes Proliferation

T cells were isolated from the spleen of C57BL/6 mice and maintained in RPMI medium (Gibco) supplemented with 10% FBS (Hyclone), 100-U/mL penicillin and streptomycin (Gibco), 1% L-glutamine (Gibco), 1% MEM non-essential amino acids, 1% MEM vitamins (Gibco), 1% pyruvate (Gibco), 0,1% B-mercaptoethanol (Gibco) (complete RPMI). To obtain naive CD4^+^ T cells, total splenocytes were labeled with antibodies to CD4 (clone RM4-5), CD62L (clone MEL-14) and CD44 (Clone IM7) and purified by FACS sorting (FacsAria-BD) (CD4^+^ CD44^low-interm^ CD62L^+^). For proliferation assays, total splenocytes were labeled with CellTrace Violet reagent (Life Technologies) and plated (2 × 10^5^ cells/well) in 96-well flat bottom plates in the presence of soluble anti-CD3 (1 μg/mL) (BD). MSC-EVs were added on day 0 and after 48 h (10^9^ particles/dose). After 72 h in culture, cells were collected and labeled with the live/dead (Life Technologies) marker and the anti-CD4 antibody for evaluation of the proliferation by FACS.

### 2.7. Differentiation of Naive CD4^+^ Cells

Naive CD4^+^ T cells were isolated by FACS sorting and plated (2 × 10^5^ cells/well) in 96-well flat bottom plates in the presence of coated anti-CD3 (2 μg/mL) (BD) and soluble anti-CD28 (1 μg/mL) (BD). For the Th0 control, no cytokines were added. For Th1 differentiation, IFN-γ (PeproTech, 10 ng/mL), IL-12 (PeproTech, 10 ng/mL) and anti-IL-4 (BD-clone 11B11) (10 μg/mL) were added. For Th17 polarization, IL-6 (Peprotech,10 ng/mL), TGF-β (R&D, 5 ng/mL), IL-23 (R&D, 10 ng/mL), anti-IFN-γ (BD—clone XMG1.2) (10 μg/mL), anti-IL-12 p40/p70 (BD—clone C17.8) (10 μg/mL), anti-IL-4 (BD—clone 11B11) (10 μg/mL) were added. For Tregs differentiation, TGF-β (R&D, 5 ng/mL), IL2 (Roche, 1 ng/mL) and anti-IFN-γ (10 μg/mL), anti-IL-12p40/p70 (10 μg/mL), anti-IL-4 (10 μg/mL) were added. To assess the direct effect on Tregs, Foxp3-GFP cells were purified by FACS sorting and maintained in culture in the presence of IL-2 (50 U/mL). For all the conditions, MSC-EVs were added at day 0 and after 48 h (approximately 10^9^ particles/dose). After 5 days, the populations of Th1, Th17 and Treg cells were analyzed by FACS (after a live/dead and CD4^+^ gate) according to the expression of IFN-γ (clone XMG1.2), IL-17 (clone TC11-18H10.1) and Foxp3 (clone MRRF-30), respectively. Th1-differentiated cells were also evaluated by confocal microscopy (Zeiss LSM 780-NLO) after labeling with anti-Foxp3 and anti-IFN-γ antibodies. Cell cultures were maintained at 37 °C with 5% CO_2_ in a humidified incubator.

### 2.8. Tregs Suppression Assay

Total splenocytes were labeled with CellTrace Violet reagent and plated (75 × 10^3^/well) in a 96-well plate. Cells obtained after differentiation for Th1, with or without MSC-EVs treatment were co-cultured in the ratios 2:1 (150 × 10^3^ cells/well), 1:1, 1:2, 1:4 and 1:8. Proliferation was stimulated with soluble anti-CD3 (1 μg/mL). As suppression control, sorting purified Tregs cells (Foxp3^+^) were used in the same ratios. Cells were maintained in complete RPMI medium for 72 h at 37 °C with 5% CO_2_ in humidified incubator. After this time, cells were collected and labeled with live/dead dye (Life Technologies) and anti-CD4 (clone RM4-5) and anti-CD8 (clone 37.51) antibodies for proliferation evaluation.

### 2.9. Flow Cytometry Analysis (FACS)

Cells were labeled with live/dead (Life Technologies) as the same time as with antibodies for surface molecules. Both the frequency and the fluorescence intensity were evaluated. For the detection of intracellular cytokines, Th1 or Th17 differentiated cells were stimulated with Phorbol 12-myristate 13-acetate (PMA) (Sigma) (50 ng/mL) (Sigma), ionomycin (500 ng/mL) (Sigma) and Golgi stop (1:1000) (BD) for 4 h at 37 °C. Cells were collected and labeled with anti-CD4 and live/dead and then intracellular labeling was performed with antibodies against cytokines and transcription factors using Transcription Factor Staining Buffer Kit (Tonbo Biosciences). In some cases, Foxp3-GFP animals were used to detect Foxp3^+^ cells directly. All data were collected on the FacsCAnto or Fortessa (BD) cytometers and analyzed by FlowJo software (Tree Star, USA).

### 2.10. Real-Time PCR (RT-PCR)

The evaluation of gene expression was performed through real-time PCR reactions. The reactions were performed on GeneAmp 7700 (Applied Biosystems-USA) using the SYBR Green and Taqman systems. The total RNA of the samples was obtained by Trizol (Life Technologies) and the complementary DNA synthesized from the messenger RNA. As endogenous control, the gene HPRT was used. The results were analyzed based on the CT (cycle threshold), using the formula 2^−ΔΔCT^ or the formula 10000/2^ΔCT^ [31].

Primers Taqman: HPRT: Mm01545399_m1, IFN-γ: Mm01168134_m1, Foxp3: Mm00475156_m1 Tbet: Mm00450960_m1.

Primers Sybr: HPRT F: CTCATGGACTGATTATGGAC, HPRT R: GCAGGTCAGCAAAGAACTTA, TGFBR2 F: CCGCTGCATATCGTCCTGTG, TGFBR2 R: AGTGGATGGATGGTCCTATTACA, PKM2 F: GCCGCCTGGACATTGACTC, PKM2 R: CCATGAGAGAAATTCAGCCGAG, HK2 F: TGATCGCCTGCTTATTCACGG, HK2 R: AACCGCCTAGAAATCTCCAGA, ACLY F: CTCCAAGAAGCCAAATCTTATC, ACLY R: ATATTCATCAGCTTCCTCCC, PDK1 F: AGGATCTGACTGTGAAGATG, PDK1 R: TGGAAGTACTGTGCATAGAG, PPP2R5E F GACGGATTTTCTCGGAAGTCC, R: GAGGTTGGAACGTCTTTCAGC, PIK3R3 F: TACAATACGGTGTGGAGTATGGA, R: GAGTCATTGGCTTAGGTGGCT.

### 2.11. miRNA PCR Array and in Silico Analysis

Total RNA of Th1-differentiated cells was isolated using miRNeasy Mini Kit (Qiagen). cDNA was synthesized from 300 ng of the mRNA using the miScript II RT kit (Qiagen). PCR array of miRNAs were run in 96-well plates for each sample (3 samples per group) following the instructions of the miScript MIMM-111Z- T Cell & B Cell Activation miRNA PCR Array (Qiagen) assay. Analysis were performed using the Qiagen website. miRNAs differentially expressed between the groups were selected and only the two miRNAs more significantly upregulated and two more significantly downregulated were considered. Three different online databases (TargetScan7 [32], miRDB [33] and Starbase [34]) were used to obtain the targets of these miRNAs. The InteractiVenn [35] website was used to select the intersections between the target genes empirically obtained from the different databases. Enrichr [36] platform was used to obtain the correlations between these target genes and possible signaling pathways in which these genes are involved. Thus, 4 signaling pathways (KEGGS pathway) that could be modulated by the miRNA expression changes were found. Analyzing the signaling pathways involved, we selected 10 of the target genes to evaluate their expression by RT-PCR.

### 2.12. Glycolytic Stress Test—Seahorse

Extracellular acidification rates (ECAR) were measured using Extracellular Flux Analyzers (Seahorse Bioscience). After Th1 differentiation, cells were plated to XF assay media without glucose. Glucose 10 mM (Sigma-Aldrich), 1-ug/mL oligomycin (Sigma-Aldrich), 22 mM 2-deoxiglicose (2-DG) (Agilent) and media were added in this order using the ports on the XF96 cartridges. The data were collected using the XF Reader software (Seahorse Bioscience). Glycolysis was calculated by the difference between ECAR rates after glucose injection and the basal rate (before glucose injection). Glycolytic capacity was calculated by the difference between ECAR rates reached after oligomycin injection and the ECAR rates reached before glucose injection. The glycolytic reserve was calculated by the difference between glycolytic capacity and glycolysis rate. Finally, ECAR rate prior to glucose injection was determined as non-glycolytic acidification.

### 2.13. Mitochondrial Membrane Potential Detection Assays

Mitochondrial membrane potential (△ψ_m_) was detected in cells after differentiation. Cells were incubated with TMRE (Abcam) (0.3 μM) or MitoTracker Deep Red FM (Life Technologies) (0.3 μM) for 30 min at 37 °C. For evaluation by FACS the cells were also labeled with live/dead and anti-CD4 and the fluorescence intensity of TMRE and MitoTracker was calculated by MFI (Median of Fluorescence). To get the images the cells were labeled concomitantly with Hoescht, washed and immediately subjected to imaging of 5 fields per well using a 40x magnification in the InCell Analyzer 2200 (GE) equipment. For quantitative analysis of fluorescence intensity and percentage of positive cells, InCell Investigator software was used. As a control, cells were incubated with CCCP (Carbonyl cyanide m-chlorophenyl hydrazone) (10 μM) for 30 min.

### 2.14. OVA Immunization In Vivo

Six-to-eight-week C57BL/6 mice were immunized with OVA protein (Ovalbumin grade V, Sigma-Aldrich). A mixture of Montanide ISA 50 V adjuvant (50%) [37] (Seppic) and OVA (200 μg) + Tween-20 (1%) + PBS or MSC-EVs (60 μL/animal) was prepared and injected at the base of the tail (200 μL/animal). After 7 days, the animals were euthanized. Inguinal and periaortic lymph nodes were collected, stimulated with PMA (50 ng/mL) and Ionomycin (500 ng/mL) and Golgi stop (1: 1000) and labeled with antibodies for the detection of IFN-γ and Foxp3 by FACS.

### 2.15. Statistical Analysis

Data were analyzed by ANOVA or Student’s t-test. All results are presented as mean and standard deviation. Values of *p* < 0.05 were considered statistically significant.

## 3. Results

### 3.1. Isolation and Characterization of MSC-EVs

**Figure 1:**
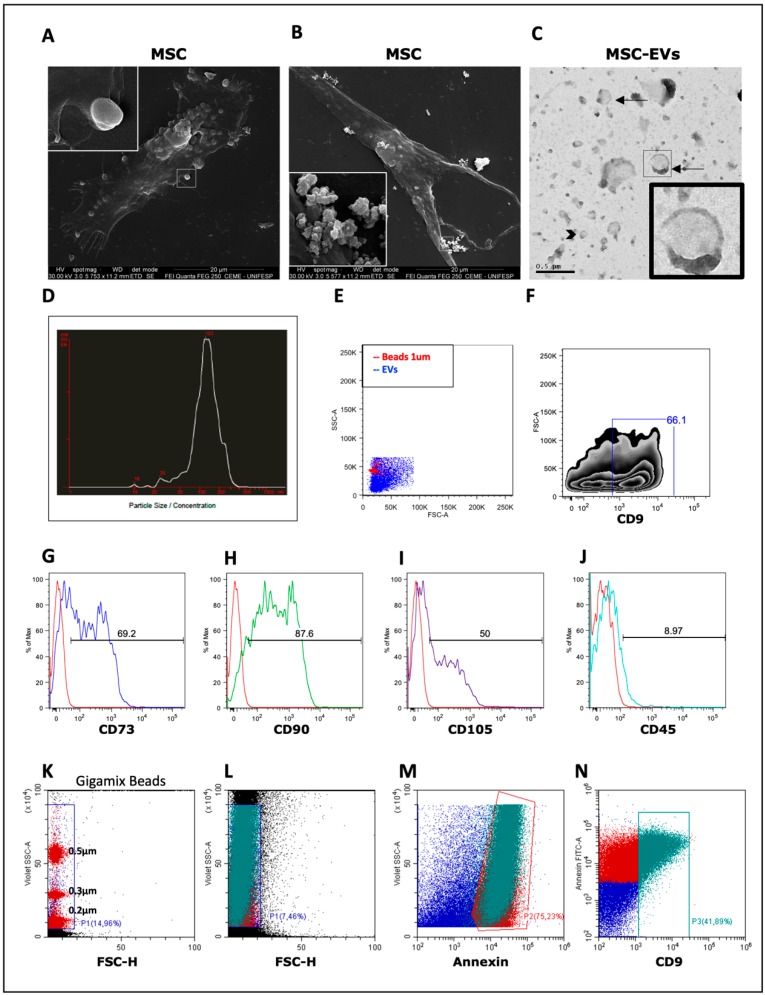
Characterization of mesenchymal stromal cell extracellular vesicles (MSC-EVs): To verify EVs releasing, mesenchymal stromal cells (MSCs) were visualized in a scanning electron microscope (**A**,**B**). The release of vesicles of different sizes was demonstrated. In A, a larger vesicle is budding from the cell membrane, while in B a pool of smaller vesicles is released. MSC-EVs were visualized in a transmission electron microscope, showing the characteristic double membrane structure. Arrows indicate larger vesicles, compatible with microvesicles, whereas arrow heads indicate smaller vesicles, compatible with exosomes (**C**). To obtain the distribution of size and concentration (particles/mL), MSC- EVs were analyzed by NanoSight (**D**). The extracellular vesicles (EVs) were analyzed by flow cytometry analysis (FACS), using 1-μm beads as reference (**E**) and labeled with anti-CD9 (**F**). Expression of present and absent markers of MSCs was also evaluated in MSC-EVs with anti-CD73, CD90, CD105 and CD45 antibodies (**G**–**J**). MSC-EVs were evaluated by CytoFLEX where they were gated based on Gigamix beads size (**K**,**L**) and stained for Annexin (**M**) and CD9 (**N**).

The adipose tissue-derived MSCs used in this study was provided by a cell bank which cells were previously characterized by our group [38]. A scanning electron microscope was used to demonstrate that MSCs release EVs of different sizes and origins (Figure 1A,B). TEM analysis showed that MSC-EVs presented a spheroid shape, with a bi-lipid membrane structure and varied sizes, representing a mixed population of smaller vesicles, that could be exosomes (approximately 100 nm) and larger vesicles, for example, microvesicles (between 100 and 1000 nm) (Figure 1C). MSC-EVs presented a mean size of 150–200 ηm with concentration, analyzed by NTA, of approximately 10^11^ particles/mL (Figure 1D). Expression of MSCs markers by EVs was detected using FACS. Calibration beads of 1 μm were used to adjust the parameters of size (FSC) and granularity (SSC) (Figure 1E). Over half of EVs expressed CD9 (Figure 1F) and the CD9^+^ particles were positive for CD73, CD90 and CD105 and negative for CD45 (Figure 1G–J), as observed at MSCs [2]. In addition, The CytoFLEX also was used to detect and better characterize the MSC-EVs. Using Gigamix beads the MSC-EVs were gated according to their corresponding size (Figure 1K,L) and they expressed classical EVs markers as annexin and CD9 (Figure 1M,N).

### 3.2. MSC-EVs are Incorporated by CD4^−^ T Cell and Alter Lymphocyte Proliferation and Differentiation

**Figure 2:**
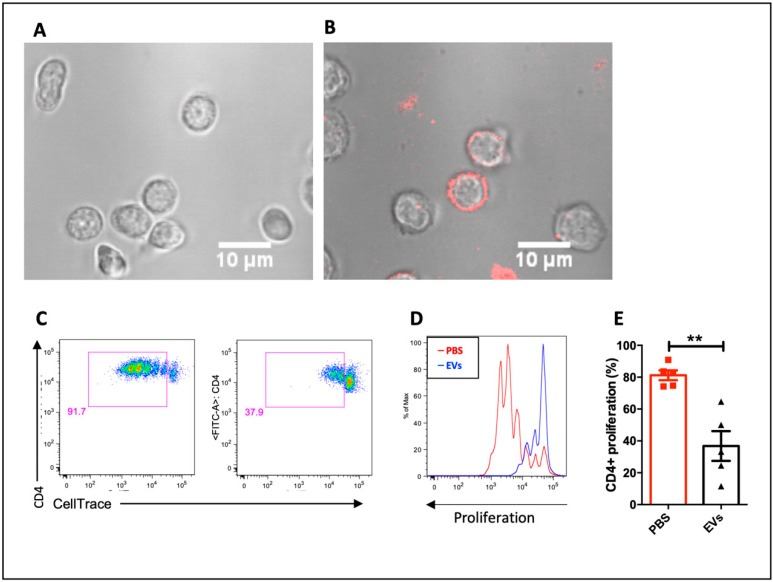
Effects of MSC-EVs on lymphocytes proliferation: incorporation of PKH26-labeled MSC-EVs by purified naive CD4+ cells using confocal microscopy, before (**A**) and 15 h after the addition of EVs (**B**). MSC-EVs effect on total splenocytes proliferation when stimulated by anti-CD3 and anti-CD28 (**C**–**E**). Data are representative of 5 independent experiments (** *p* < 0.01).

To investigate the biodistribution of MSC-EVs, confocal microscope assay was performed and the MSC-EVs were visually incorporated by CD4^+^ T lymphocytes (Figure 2A,B). In order to evaluate the functional effect of the MSC-EVs on lymphocytes proliferation, total splenocytes were labeled with Cell trace violet and stimulated with anti-CD3. The proliferation was evaluated by FACS 3 days after the stimulus. MSC-EVs were able to reduce the proliferation of activated CD4 T lymphocytes by approximately 50% (Figure 2C–E).

**Figure 3:**
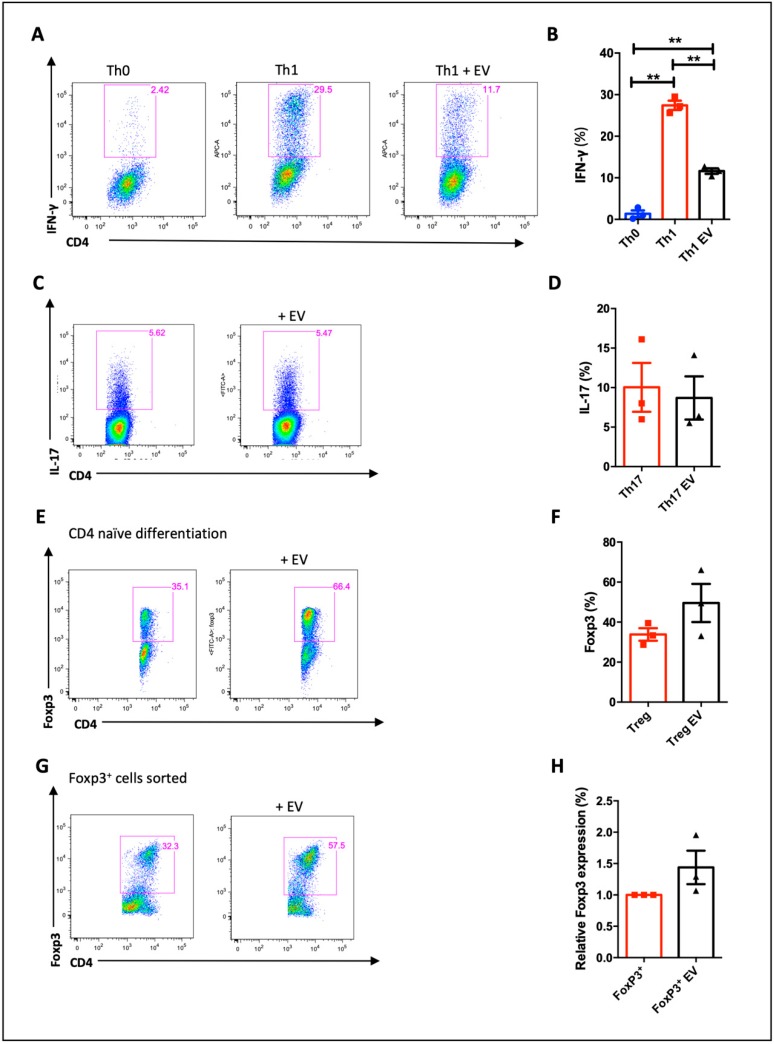
Effect of MSC-EVs on differentiation for T helper subsets: purified CD4 cells were differentiated for Th1 (**A**,**B**) Th17 (**C**,**D**) and Treg (**E**,**F**) and evaluated by detection of IFN-γ, IL-17 and Foxp3, respectively, by FACS. Foxp3+ cells were purified from spleen by FACS sorting and maintained in culture in the presence of IL-2 and MSC-EVs. Foxp3 expression was evaluated after 5 days in culture (**G**,**H**). Data are representative of 3 independent experiments (** *p* < 0.01).

Since we observed that MSC-EVs inhibited CD4^+^ T cell proliferation, we next sought to assess whether the MSC-EVs would influence on T cell differentiation. Then, we sorted naïve CD4^+^ T cells and polarized them to differentiate to Th1, Th17 and Treg cells in the presence of MSC-EVs. The presence of MSC-EVs significantly affected Th1 differentiation as observed by the decreasing of IFN-γ production (Figure 3A,B), although nothing was observed regarding the IL-17A production in the presence of MSC-EVs (Figure 3C,D). In the Treg differentiation, it was detected higher numbers of Foxp3^+^ cells within MSC-EVs-treated group when compared to untreated ones, however without statistical differences (Figure 3E,F). Further, to access the precise effect of MSC-EVs in differentiated Tregs, we purified mature Tregs (Foxp3^+^GFP^+^) by cell sorting from spleen and lymph nodes of FOXP3-GFP knock in mice and stimulated them with plated-bound anti-CD3 and soluble anti-CD28 in the presence of MSC-EVs. Again, we did not observe any statistical improvement of the *foxp3* expression, even with higher number of Foxp3^+^ cells detected in the MSC-EVs-treated group (Figure 3G,H). Thus, these results altogether indicate that MSC-EVs can modulate the Th1 differentiation.

### 3.3. MSC-EVs Induce Foxp3 Expression in Th1 Differentiated Cells

**Figure 4:**
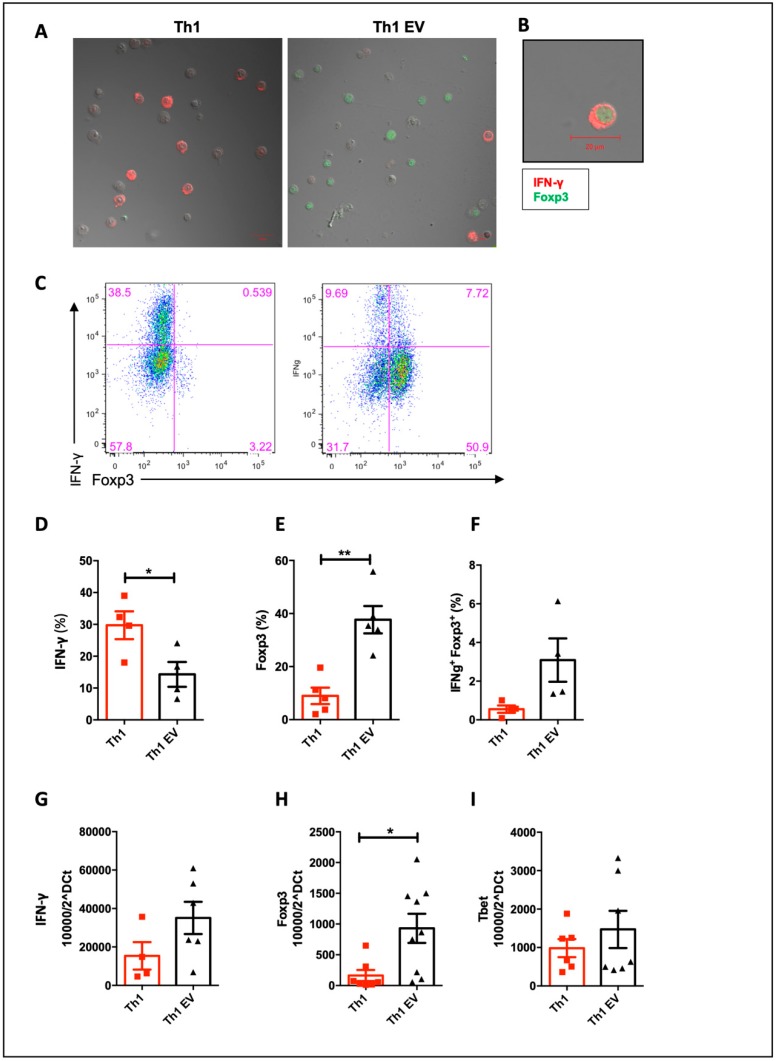
Effects of MSC-EVs on Th1 differentiation: Purified naive CD4^+^ cells were differentiated for Th1 and IFN-γ and Foxp3 were detected by confocal microscopy (**A**), FACS (**C**–**F**) and RT-PCR (**G**,**H**). Detection of IFN-γ (red) and Foxp3 (green) by confocal microscopy; (**A**) demonstrates the presence of double positive cells for Th1 cytokine and Tregs transcription factor Foxp3 (**B**,**F**). Expression of Tbet transcription factor was also evaluated by RT-PCR (**I**). Data are representative of 4 independent experiments (* *p* < 0.05).

In order to understand how MSC-EVs inhibited Th1 differentiation we decided to investigate the generation of Foxp3^+^ cells during Th1 differentiation in the presence of MSC-EVs, since this is one defined mechanisms of MSCs immune regulation [6,8,9]. As expected, the addition of MSC-EVs decreased IFN-γ production visualized by reduced number of red dye-labeled cells (Figure 4A,C,D). Notably, MSC-EVs addition in CD4^+^ naïve T cells polarized to Th1 increased the frequency Foxp3^+^ cells and expanded Foxp3 expression (green dye-labeled cells) (Figure 4A,C,E). This finding of increase in Foxp3^+^ cells in Th1 cells in the presence of MSC-EVs was confirmed by confocal microscopy and by flow cytometry (Figure 4A,B). The addition of MSC-EVs increased the frequency of Foxp3^+^ cells (Figure 4C,E). Surprisingly, IFN-γ and Foxp3 double-positive cells were also identified after treatment with MSC-EVs (Figure 4A,B,F).

Expression of IFN-γ and Foxp3 was also evaluated by RT-PCR, as well as Tbet (Th1-specific transcription factor) expression. Although a reduction of IFN-γ by FACS and confocal microscopy was demonstrated, we did not see a difference in the expression of IFN-γ mRNA (Figure 4G). The same was observed for Tbet expression (Figure 4I). However, the Foxp3 increase was confirmed by real-time PCR analysis (Figure 4H).

**Figure 5:**
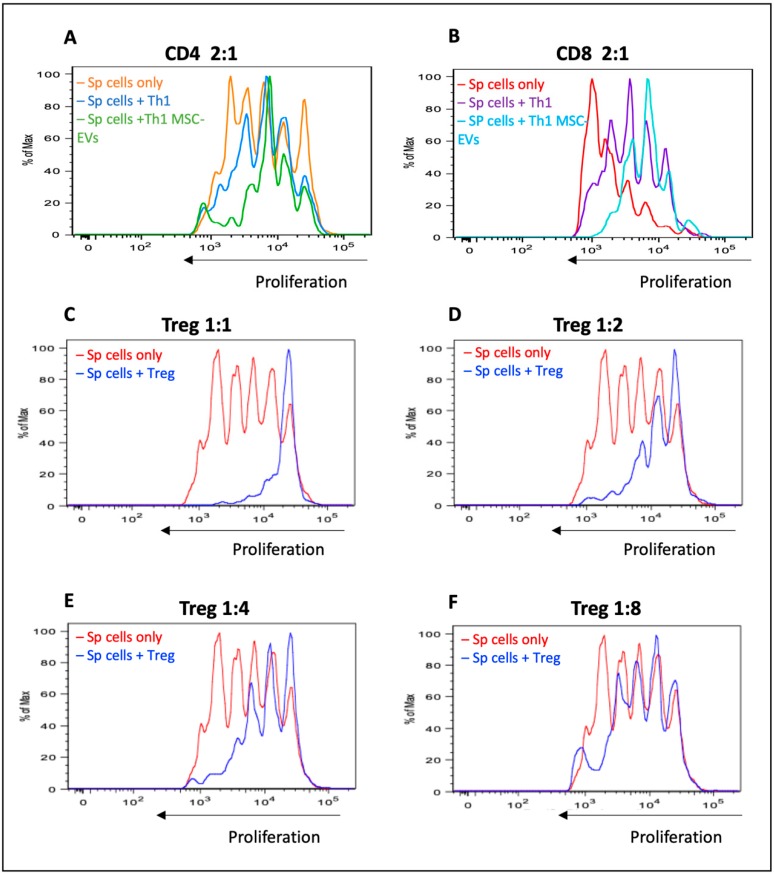
Functional analysis of the Foxp3^+^ cells generated. A suppression assay using Th1 differentiated cells, treated or not with MSC-EVs, in co-culture with total splenocytes was performed. The effects on the proliferation of CD4^+^ (**A**) and CD8^+^ T (**B**) cells in the 2:1 ratio were shown. Tregs cells were used as a positive control of CD4^+^ T cell suppression in several ratios (**C**–**F**). Data are representative of 2 independent experiments (* *p* < 0.05).

In an attempt to confirm functional regulatory profile of these MSC-EVs-modulated T cells generated, we performed a suppression assay using differentiated Th1 cells in the presence or absence of MSC-EVs in co-culture with total splenocytes at different ratios (Figure 5). As control, Tregs cells were purified by sorting and co-cultured with total splenocytes, also in different ratios (Figure 5C–F). In the 2:1 ratio, we observed that MSC-EVs-modulated T cells (Foxp3^+^ IFN-γ^+^) in the presence of MSC-EVs were able to suppress CD4^+^ T cell (Figure 5A) and CD8^+^ T (Figure 5B) proliferation. The suppressive effect of these cells was similar to Tregs at 1:4 ratio (Figure 5E).

These results suggest that cells differentiated to Th1, in the presence of MSC-EVs, are reprogrammed to a more regulatory profile, decreasing the frequency of IFN-γ producing cells and increasing Foxp3 expressing cells, which are able to suppress the proliferation of total splenocytes.

### 3.4. The Global Analysis of miRNA Array Reveals Possible Targets of miRNA Regulation in Lymphocytes Treated with MCS-EVs

**Figure 6:**
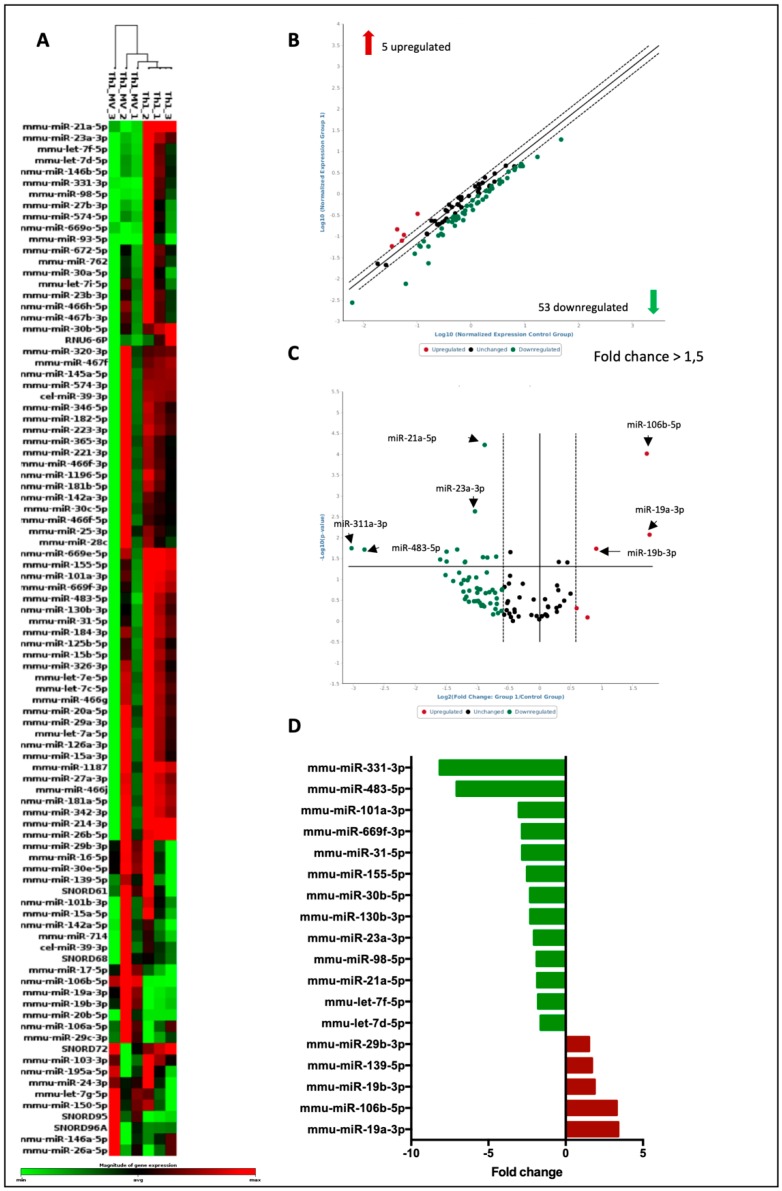
PCR array of miRNAs expressed from Th1 differentiated cells: Comparative heat map of miRNAs in MSC-EVs treated and untreated groups (**A**). Graphs representing differentially expressed miRNAs with fold change > 1.5 (**B**) and differentially expressed miRNAs with significant variation (*p* < 0.05) (**C**). Bar graph demonstrating the miRNAs with the highest variations (**D**). *n* = 3 (* *p* < 0.05).

**Figure 7:**
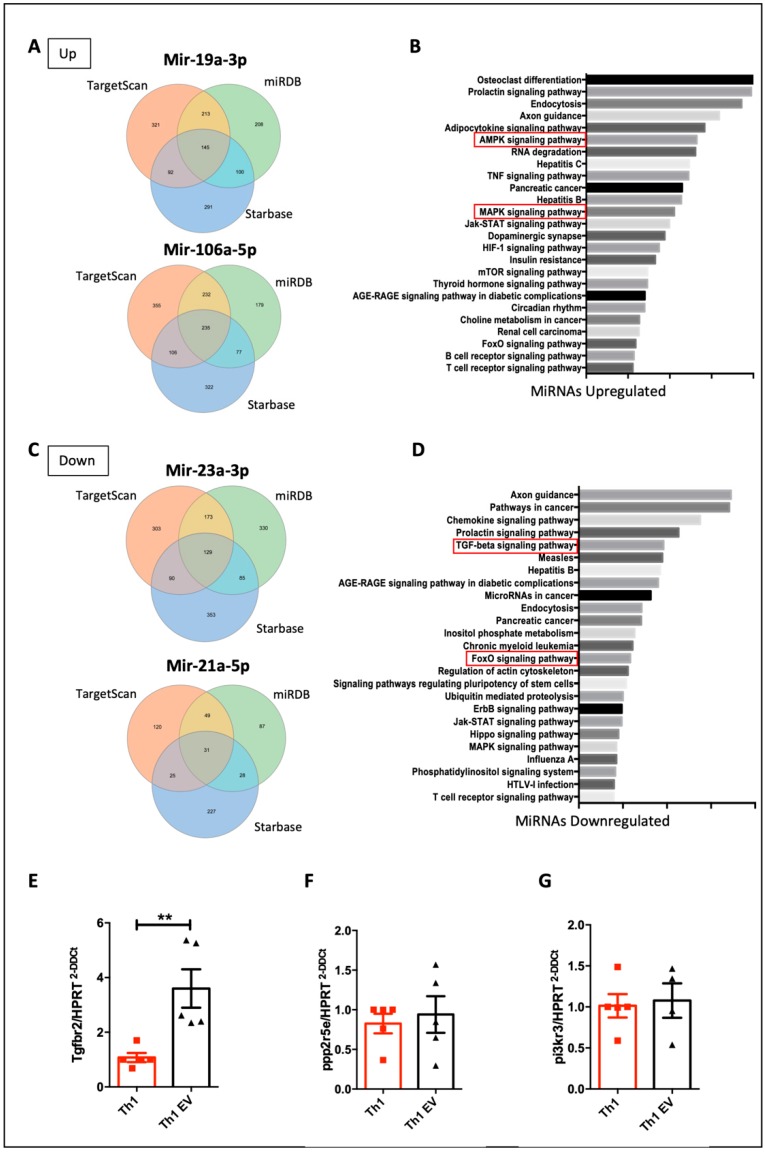
In silico analysis of signaling pathways regulated by miRNAs: Venn diagrams representing the intersection of upregulated (**A**) and downregulated (**C**) miRNAs found in 3 different databases. Biologic processes related to the most overexpressed (**B**) and the most reduced (**D**) miRNAs found by KEGGS pathway analysis on the Enrichr platform. The expressions of TGFBR2 (**E**), PPP2R5E (**F**) and PI3KR3 (**G**) were detected by RT-PCR. *n* = 5 (** *p* <0.01).

The transfer of miRNA has been considered the main mechanism by which EVs exert their effects [39]. Therefore, in order to detect differentially expressed miRNAs in Th1 cells and possible molecular signature involved in the Th1 regulation by MSC-EVs, a specific platform for detection miRNAs associated with signaling pathways in B and T cell activation (QIAGEN) was used. This kit comprises a panel with 84 miRNAs involved in the differentiation of lymphocytes (Figure 6A). We detected 5 upregulated and 53 downregulated miRNAs in T cells differentiated into Th1 in the presence or not of MSC-EVs (fold change> 1.5) (Figure 6B). Further, we verified the most regulated transcript and only 3 miRNAs had significantly its expression decreased and 13 showed to be statistically increased (*p* < 0.05) (Figure 6C,D). In an attempt to detect signaling pathways and genes regulated by these differentially expressed miRNAs, an in silico analysis was carried out, in which the most modulated miRNAs were addressed (upregulated: miR-19a-3p and miR106a-5p and downregulated: miR23a-3p and miR-21a-5p). The target genes of each miRNA were found in 3 different and independent databases (Figure 7A,C). Putative target genes commonly found in these 3 databases were analyzed in the ENRICHR website to detect possible signaling pathways involved (Figure 7B,D). Among the pathways regulated by the positively regulated miRNAs (pathways that would be less active), the AMPK and MAPK pathways were selected (Figure 7B and Table S1), whereas the pathways regulated by reduced miRNAs (pathways that would be more active), the TGF-β pathway and FoxO pathway were selected (Figure 7D and Table S2). The target genes that participate in each pathway analyzed were identified and some genes were validated by RT-PCR (Tables S1 and S2). TGFBR2 expression was the only gene evaluated which had its expression regulated (Figure 7E–G).

Expression of AMPK pathway genes PPP2R5E (Figure 7F) and PI3KR3 (Figure 7G), which are target genes of the upregulated miR-19a-3p, was not altered. Since TGFBR2 is regulated by miRNA 23a-3p, which was downregulated in the presence of MSC-EVs, its expression was increased in the MSC-EVs treated group (Figure 7E). These results confirm, in part, the participation of miRNAs in Th1 cell modulation by MSC-EVs and suggests that TGF-β signaling pathway (miRNA-23a-3p/TGFBR2) may modulate Th1 differentiation.

### 3.5. Treatment with MSC-EVs Alters the Metabolism of Differentiated T Cells to Th1, Decreasing Mitochondrial Membrane Potential and Glycolysis

**Figure 8:**
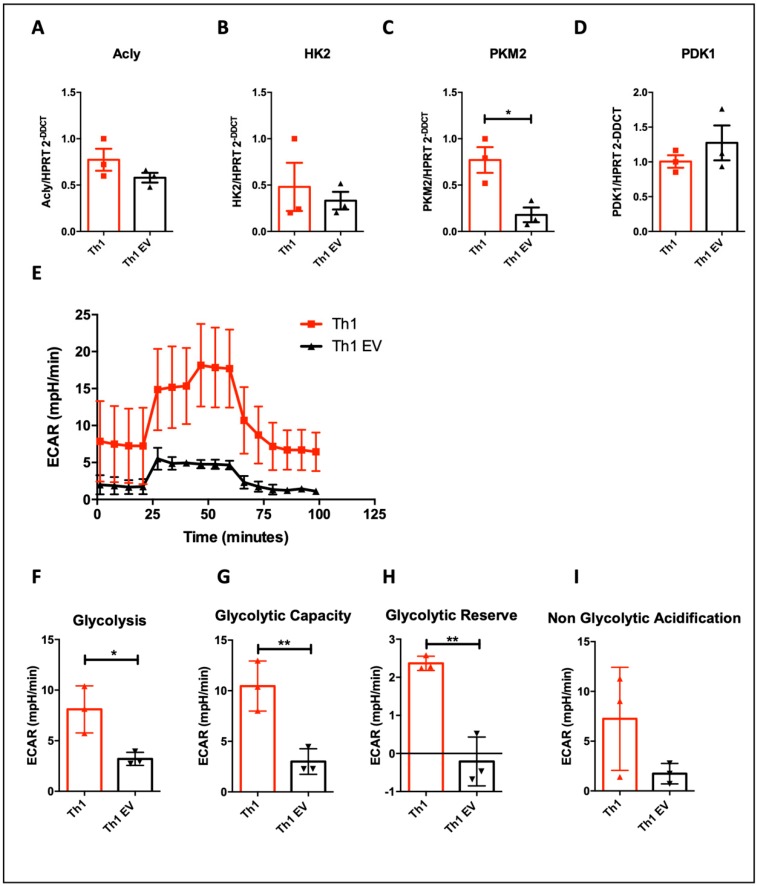
Effects of MSC-EVs on T cell glycolytic metabolism: Th1-differentiated cells were evaluated by the expression of metabolic-related genes, such as ACLY (**A**), HK2, (**B**), PKM2 (**C**) and PDK1 (**D**) by RT-PCR. A Seahorse analysis was performed in order to evaluate glycolytic metabolism (**E**). Separately, glycolysis (**F**), glycolytic capacity (**G**) glycolytic reserve (**H**) and non-glycolytic acidification (**I**) rates were obtained. *n* = 3 (* *p* < 0.05).

It is well known that TGF-β pathway can regulate metabolic process through the regulation of mTOR pathway, which can regulate T cell metabolism. Therefore, in order to further investigate possible determinant mechanisms by which MSC-EVs exert effects on T lymphocytes, as a consequence TGF-β pathway modulation observed in the miRNA profile, we searched for specific metabolic and mitochondrial changes in T cells. Genes involved in metabolic regulation (ACLY, HK2, PKM2 and PDk1) were evaluated (Figure 8A–D) and PKM2 showed reduced expression after treatment with EVs (Figure 8C), while no statistical difference was observed in the other molecules evaluated.

Th1 cells depend on glycolysis to produce IFN-γ [40,41]. On the other hand, inhibition of glycolysis favors Treg differentiation [40]. Therefore, we performed a Seahorse analysis in order to obtain the extracellular acidification rate (ECAR), for direct measurement of glycolysis index. A prominent decreasing in ECAR rate in the cells differentiated in the presence of MSC-EVs was observed (Figure 8E). In addition, we detected several metabolic parameters decreased in T cells treated with MSC-EVs, as glycolysis rate (Figure 8F), glycolytic capacity (Figure 8G) and glycolytic reserve (Figure 8H), considering that no statistical difference was seen at non-glycolytic acidification (Figure 8I) rate. All these data confirm that T cells are less glycolytic after MSC-EVs addition and this may be affecting their IFN-γ production.

**Figure 9:**
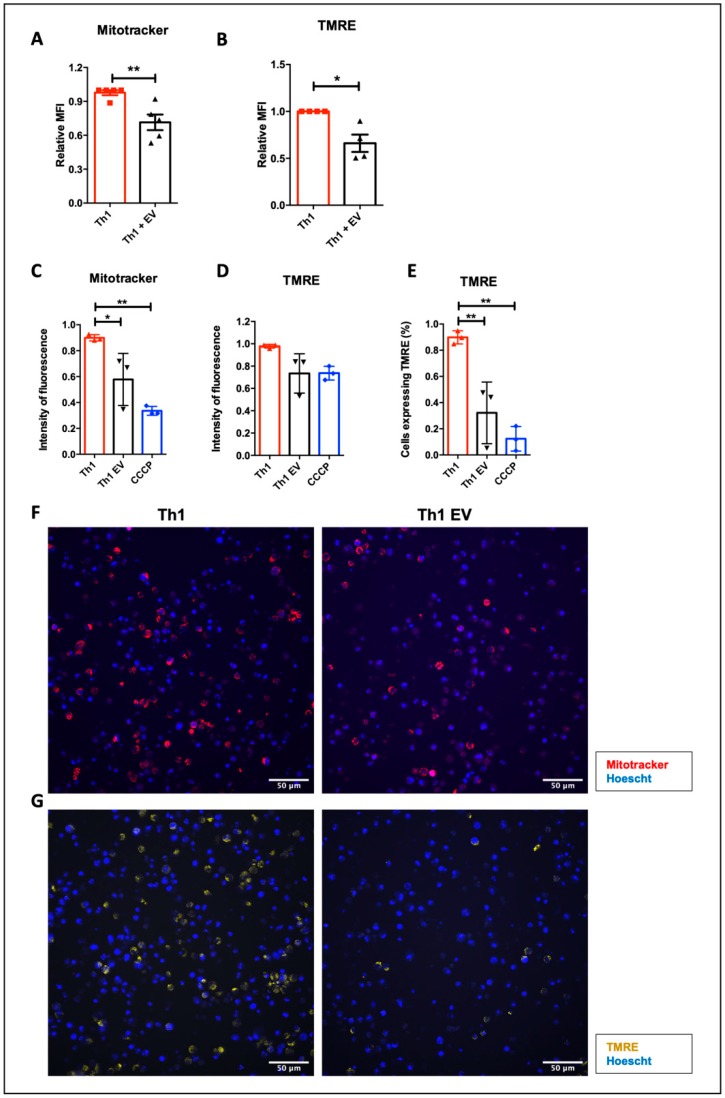
Effects of MSC-EVs on mitochondrial metabolism: The mitochondrial membrane potential (△ψm) was evaluated using the specific markers TMRE and MitoTracker Deep Red. The fluorescence intensity of MitoTracker and TMRE was detected by flow cytometry (**A**,**B**) and by images using InCell Analyzer (**C**,**D**). The percentage of cells expressing TMRE was detected by microscopy (**E**). Representative images of Th1 cells differentiated in the presence or not of MSC-EVs stained for MitoTracker in red (**F**) or TMRE in yellow (**G**). Data are representative of 4 independent experiments (FACS) and 3 independent experiments (InCell). * *p* < 0.05 (** *p* < 0.01).

To verify whether MSC-EVs can further impact mitochondrial metabolism, lymphocytes were labeled with TMRE and MitoTracker and analyzed by FACS and microscopy (InCell Analyzer). T cells treated with MSC-EVs demonstrated a decrease in mitochondrial membrane potential (△ψ_m_), represented by the reduced TMRE and MitoTracker fluorescence intensity when evaluated by FACS (Figure 9A,B) and by low frequency of TMRE-positive cells when evaluated by microscope (Figure 9E,G). Besides no difference in intensity of TMRE fluorescence (Figure 9D,G), this was confirmed by the decrease of MitoTracker fluorescence (Figure 9C,F). As a control, CCCP, a decoupling agent inhibiting oxidative phosphorylation, was added and a △ψ_m_ reduction was observed.

These results suggest that MSC-EVs can regulate metabolic pathways in Th1 differentiated cells, which may also be related to the reduction of IFN-γ and generation of Foxp3^+^ cells and to the modulation of the TGF-β pathway.

### 3.6. MSC-EVs Treatment Expands Tregs In Vivo

**Figure 10:**
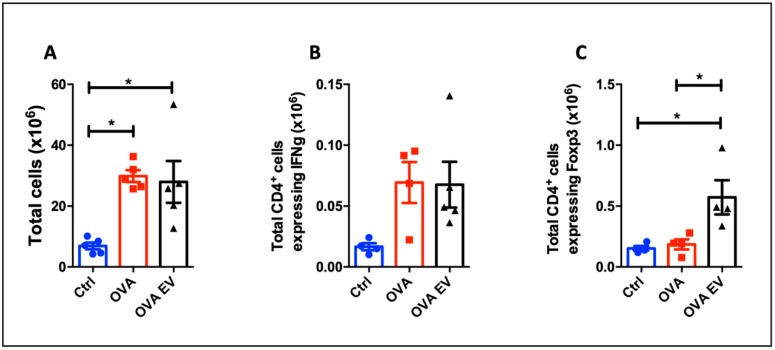
Effects of MSC-EVs in vivo: C57Bl/6 mice were immunized with OVA and treated with MSC-EVs. After 7 days, the animals were euthanized, and the draining lymph nodes were collected. The total number of cells in the lymph nodes (**A**) and the total number of CD4 cells producing IFN-γ (**B**) and expressing Foxp3 (**C**) were evaluated. *n* = 5 (* *p* < 0.05).

To verify the regulatory effects of MSC-EVs on Th1 differentiation in vivo, we choose a murine immunization model with OVA. After 7 days, the animals were euthanized, and the draining lymph nodes were collected for analysis. It was detected that immunization with OVA significantly increased the number of infiltrating cells in the lymph nodes (Figure 10A). After 7 days, the number of CD4^+^ T cells expressing IFN-γ after immunization showed a tendency to increase and no effect was observed after MSC-EVs injection (Figure 10B). On the other hand, the number of Foxp3-expressing CD4^+^ T cells was dramatically elevated after treatment with MSC-EVs (Figure 10C). Finally, these results confirm the ability of MSC-EVs to expand Foxp3^+^ T cells during an inflammatory response in vivo, as it was seen in the in vitro assays.

## 4. Discussion

In this study, we demonstrate the ability of MSC-EVs to regulate Th1 cells potentially via modulation of miRNA profile associated TGF-β pathway and metabolism shifting. MSC-EVs reduced differentiation to Th1, generating cells that express Foxp3 with reduced IFN-γ production and increased suppressive capacity. The addition of MSC-EVs changed miRNAs expression in the Th1 cells, increasing TGFBR2 expression, as a consequence of the reduction of mir-23a-3p. The regulation of TGF-β pathway can be related with the regulation of metabolic pathways. Accordingly, we showed a reduction in glycolytic and mitochondrial metabolism in the cells differentiated in the presence of MSC-EVs. Our data confirm the capacity of MSC-EVs in regulating T cells and open new perspectives to the use of EVs as a therapeutic alternative to MSCs.

During EVs isolation, we excluded the larger vesicles and possible cellular debris of our MSC-EVs population by centrifuging the cell supernatant at 2.000× *g*. After centrifugation at 100.000× *g*, we obtained a mixture of EVs with the presence of exosomes, exomeres and MVs. Opting for not do the 10,000× *g* ultracentrifugation, we don’t differentiate medium size particles from smaller size ones [42]. Accordingly, the presence of different vesicle sizes was detected by scanning and transmission electron microscopy and NTA. Analysis of size distribution by NTA suggests, however, a predominant presence of smaller particles. Although several studies indicate that different fractions of EVs may have different [43] or even opposites effects, [44,45], the choice of using this mixture of particles is mainly because EVs may be released simultaneously by the cells. Additionally, the yield of EVs from MSC culture supernatant is not high and large amounts of particles from purified EVs are needed to obtain modulatory effect. The mixed population of EVs obtained showed the molecules from parental cells, expressing MSCs [27,46] markers, and were mostly positive for Annexin and CD9, classical EVs markers.

PKH dyes have been widely used for labeling extracellular vesicles [47,48], however, their use has been controversial. Recent studies have shown that nanoparticles of dye can also be internalized [49,50], but in a lower extension as EVs-labeled particles [49]. When we evaluated the incorporation of MSC-EVs by lymphocytes using PKH26, we found an accumulation of EVs on the cell surface. Although it is not possible to differentiate whether there was accumulation in the cytosol or fusion with the plasma membrane, studies have suggested that EVs naturally associate with the lymphocyte membrane [43,51] exerting their modulatory effects. In this sense, the addition of MSC-EVs significantly reduced the proliferation of T cells. The literature is controversial regarding the effects of MSC-EVs on lymphocyte proliferation. Some studies have shown inefficiency of MSC-EVs in inhibiting T cell proliferation [52,53,54], others have demonstrated lower efficiency of EVs when compared to MSCs [55,56,57,58] while others have reported ability to inhibit lymphocyte proliferation [59,60,61]. The difference between these studies may be due to variations in the dose and types of EVs and T cell sources used.

Functionally, authors have reported that MSC suppression on T-cell proliferation is more related to cell cycle inhibition than apoptosis induction [62]. To confirm that our EVs were not killing the cells, we performed live/dead labeling by cytometry and did not see changes in cell viability following the addition of MSC-EVs in our culture conditions (Figure S1). In order to evaluate whether the MSC-EVs could alter specifically T cell response, we differentiated naïve CD4^+^ T cells into different T cell subtypes: Th1, Th17 and Treg. It was previously shown that MSCs or MSC-EVs can alter the balance between different T cell subtypes [63,64]. In a study using PBMC cells from type I diabetes patients, MSC-EV administration decreased Th17 response while increased Treg response [65]. Based on that, we differentiated lymphocytes to a pro-inflammatory condition (Th1) and as it was shown previously with MSCs treatment [66,67], a significant reduction of IFN-γ-secreting cells was observed after treatment with MSC-EVs. We then differentiated to another pro-inflammatory condition, Th17, but we did not see effect on T cell differentiation. When we evaluated the effect over Treg differentiation, we detected a trend to increase the percentage of cells expressing Foxp3. The same was observed when we evaluated the effects directly on purified Tregs from the spleen. The different behavior of EVs in each condition may indicate a dependency of a favorable environment for them to perform their adequate functions, which we believe being preferentially associated with inflammatory microenvironment. For instance, in a model of skin transplantation, MSC-EVs expanded Tregs and increased animals survival only in animals that received the graft [68]. Accordingly, the presence of cytokines may influence the effects of MSC-EVs. One study observed that the presence of TNF along with EVs may promote greater effect on target cells than using EVs or TNF alone, suggesting that they may act synergistically [69]. Therefore, cytokines present in a Th1 condition, such as IL-12 or IFN-γ, may have intensified the effects of MSC-EVs.

In addition, knowing the ability of MSCs to induce Tregs [9,66,70], we also sought to evaluate the expression of Foxp3 transcription factor in T cells under inflammatory conditions. Interestingly, the addition of MSC-EVs induced a significant increase in Foxp3 expression in Th1-differentiated cells, suggesting that MSC-EVs can induce a regulatory transcriptional profile in Th1-type cells. In our study, the reduction of Tbet expression was not demonstrated after the addition of MSC-EVs. However, the co-expression of Tbet and Foxp3 transcription factors, by itself, may suggests the induction of more regulatory cells. Recent studies have demonstrated the existence of a type of hybrid cells that co-express transcription factors of different T subtypes, exerting preferentially an anti-inflammatory effect [71,72,73]. It was demonstrated that the expression of Tbet in Tregs (Foxp3^+^) is important for these cells to regulate Th1 response and, consequently, regulate IFN-γ production [71,74]. Tregs can also produce IFN-γ as a way to regulate the inflammatory response [58] or generate operational tolerance [75]. Accordingly, the co-expression of Foxp3 and IFN-γ in MSC-EVs treated cells, suggests the generation of a different hybrid cell subset that may have regulatory function. In this context, the suppression assay of CD4^+^ and CD8^+^ T cell proliferation confirmed that these generated Foxp3^+^ IFN-γ^+^ T cells are functional and have regulatory profile. Therefore, the treatment with MSC-EVs generate a rare subtype of Th1 cells that at same time that produce IFN-γ and express the regulatory transcription factor Foxp3, which make them acquire regulatory properties, regulating lymphocyte proliferation. However, the molecular mechanisms of how these cells behave should be further investigated.

In order to investigate possible mechanisms involved in the MSC-EVs effects over Th1 cells, the PCR miRNA array platform associated to T and B cell activation pathways was used as a tool to identify miRNAs and signaling pathways involved in the regulation of Th1 response. We observed that MSC-EVs addition reprogrammed T cell miRNAs profile as observed during modulation of the immune system upon activation [76,77]. In silico enrichment analysis, focusing on the target genes of the most up or downregulated miRNAs, showed possible signaling pathways involved in this process. Among the pathways linked to downregulated miRNAs was observed the TGF-β pathway, which is involved in the generation of Tregs through the binding of transcription factors in the regulatory elements at the Foxp3 locus [78,79]. Therefore, we looked for genes that activate these pathways and we detected difference in *TGFBR2* (TGF-β receptor 2) expression, a gene targeted by miR-23a-3p. This receptor has important role in the control of Th1 cells. Its absence in T cells led to a more inflammatory condition, with greater production of IFN-γ by CD4^+^ T cells [80,81]. Additionally, expression of a dominant negative form of this receptor also decreased the suppressive capacity of Tregs [82]. These data suggest that the regulation of miRNAs expression and their target genes can be one mechanism by which MSC-EVs are modulating Th1 cells. Specifically, the miRNA regulation of TGFBR2 expression may be regulating IFN-γ production from Th1 cells after MSC-EVs addition.

TGF-β pathway is also involved in the inhibition of mTOR pathway, which regulates metabolic process in immune cells, favoring anabolic processes and promoting glycolysis [83]. Recently, the correlation between metabolism and immune cells has been more explored. After activation, Naïve T cells need substrates for the biosynthesis of proteins, lipids and nucleic acids essentials for cell proliferation and, hence, to accelerate this process, they switch their metabolic profile to anabolic state, mainly dependent on aerobic glycolysis [84]. This process, known as the Warburg effect [85], occurs when the cell performs glycolysis even in the presence of oxygen. Specifically, for Th1 cells, the glycolytic pathway appears to be the most important. IFN-γ production is regulated by GAPDH, a glycolytic pathway enzyme [86] and Glut-1 [41]. In contrast, Tregs is dependent mainly of the metabolism of lipids (beta oxidation), which occurs in the mitochondria [84,87] and the inhibition of glycolysis favors Treg differentiation [40]. Therefore, in order to verify if MSC-EVs treatment and its consequent signal trough TGF-β pathway could alter T cell metabolism, we evaluated the expression of genes associated with different metabolic pathways. Hexokinase 2 (HK2) is an enzyme that catalyzes the first step of glycolysis, ATP citrate lyase (ACLY) is an important enzyme in lipid biosynthesis [88], Pyruvate dehydrogenase kinase 1 (PDK1) is an enzyme that phosphorylates and inactivates pyruvate dehydrogenase, the enzyme responsible for the first step of citric acid cycle—TCA [89]—and pyruvate kinase isozyme 2 (PKM2) catalyzes the last glycolysis reaction [90]. Although we have not identified any significant differences in the expression of ACLY, PDK1 and HK2, we cannot affirm that there is no metabolic changes in T cells treated with MSC-EVs, since the regulation of these enzymes may act principally at the protein level [91,92,93]. However, importantly, we detected a difference in PKM2 expression. This enzyme has been related to the Warburg effect, especially in cancer cells [94]. PKM2 has also functions not related to metabolism, being associated with activation of inflammatory response [95], mainly acting as co-activator of HIF-1α [96,97]. Accordingly, it is demonstrated that under normoxia conditions, the absence of HIF-1α increase Tregs differentiation [40]. Finally, PKM2 can act as a regulator of mTOR pathway. A recent study has shown that the reduction of PKM2 inhibits PI3K/Akt [98] signaling, which may also justify the reduction of IFN-γ in cells treated with MSC-EVs. Additionally, when we evaluated specifically the glycolytic metabolism, a reduction of ECAR, glycolysis and glycolytic capacity and glycolytic reserve was shown, corroborating with PKM2 expression levels reduced. These data also suggest that the metabolic switch required to cytokine production was not achieved and may explain the IFN-γ reduction observed.

To further investigate the effects of MSC-EVs over T cell metabolism, we looked at mitochondrial metabolism through the detection of mitochondrial membrane potential (△ψ_m_). The evaluation of membrane potential of Th1-differentiated cells demonstrated a reduction in cells treated with MSC-EVs, which suggests a lower participation of mitochondrial metabolism in this process. Mitochondrial metabolism results in the generation of ROS (reactive oxygen species), which is important for the optimal activity of NFAT and NF-kB [99,100], involved in the signaling for T cell activation and cytokine production [101], such as IFN-γ [102]. In this context, the addition of MSC-EVs may have altered the metabolism of the T cells, reducing their activation and IFN-γ production. Additionally, in a recent study, Sukumar and colleagues isolated cells differentiated for Th1 in vitro, sorting them according to the mitochondrial potential. Cells with high △ψ_m_ produced four times more IFN-γ compared to low △ψ_m_ cells and cells with low △ψ_m_ showed a reduction in mTORC1 activity [103]. In another study, the addition of TGF-β reduced glycolytic metabolism in thymus Tregs, as a consequence of the reduction of mTOR pathway activation [104]. These data corroborate our findings that cells producing less IFN-γ, after treatment with MSC-EVs, have lower △ψ_m_ and may also suggest the participation of TGF-β pathway in this regulation.

Finally, to evaluate the MSC-EVs effect in vivo, we used an OVA immunization experimental model. Since we believe that the major effect of EVs depends on an inflammatory environment, we looked for a model that would give us a Th1 response. Therefore, we used Montanide ISA adjuvant [37], which induces predominantly a Th1 response [105]. According to what was discussed above, we believe that MSC-EVs effects are dependent on the presence of specific cytokines related to the Th1 response. When MSC-EVs were injected concomitantly with OVA, an increase in the total number of cells expressing Foxp3 was seen, confirming the ability of MSC-EVs to generate regulatory cells expressing Foxp3—as observed in vitro in a Th1 differentiation. However, we did not observe any effect on the IFN-γ production in this context. Increasing the dose may lead to a more pronounced effect. Nevertheless, these data corroborate our in vitro studies and demonstrate the ability of MSC-EVs to induce Foxp3^+^ cells under inflammatory conditions in vivo.

In summary, in this report we demonstrated that MSC-EVs have the capacity to modulate lymphocytes. We verified that Th1 cells reduced the production of IFN-γ—and surprisingly—they started to express Foxp3. As part of their regulatory mechanism, we have shown that MSC-EVs induced modifications in the miRNA profile, decreasing miR-23a-3p expression and increasing the expression of its target gene, TGFBR2, suggesting active participation of TGF-β pathway in this regulation. This pathway, in turn, may inactivate the mTOR pathway and, as a consequence, alter the metabolic profile of T cells treated with MSC-EVs. These cells presented a reduction in the glycolytic metabolism, as well in the mitochondrial metabolism, what can be related to PKM2 modulation. This molecular crosstalk can explain the presence of less activated Th1 cells with lower IFN-γ production. Concluding, our findings suggest that MSC-EVs are able to specifically modulate activated T cells at an alternative regulatory profile by miRNAs and metabolism shifting. Thus, we can speculate that MSC-EVs can induce immunological tolerance, in vivo, contributing for their future use as an alternative therapy.

## Supplementary Materials

The following are available online at https://www.mdpi.com/2073-4409/9/4/1059/s1, Table S1. Upregulated miRNAs’ target genes selected for analysis and their corresponding signaling pathways. Table S2. Downregulated miRNAs’ target genes selected for analysis and their corresponding signaling pathways. Figure S1. Cells viability after MSC-EVs treatment: Live and dead staining of cells differentiated for Th1 after MSC-EVs treatment. Data representative of 4 independent experiments. Video S1. Animation of incorporation of PKH26-labeled MSC-EVs by purified naive CD4+ cells: Images were captured at 15 min interval over the course of 15h and collated in sequential order. See Figure 2A,B.
