# Supplementary figures and images for: Extracellular Vesicles isolated from Mesenchymal Stromal Cells Modulate CD4+ T Lymphocytes Toward a Regulatory Profile
Source: Cells. 2020 Apr 23;9(4):1059. doi: 10.3390/cells9041059 (PMC7226573; doi:10.3390/cells9041059)

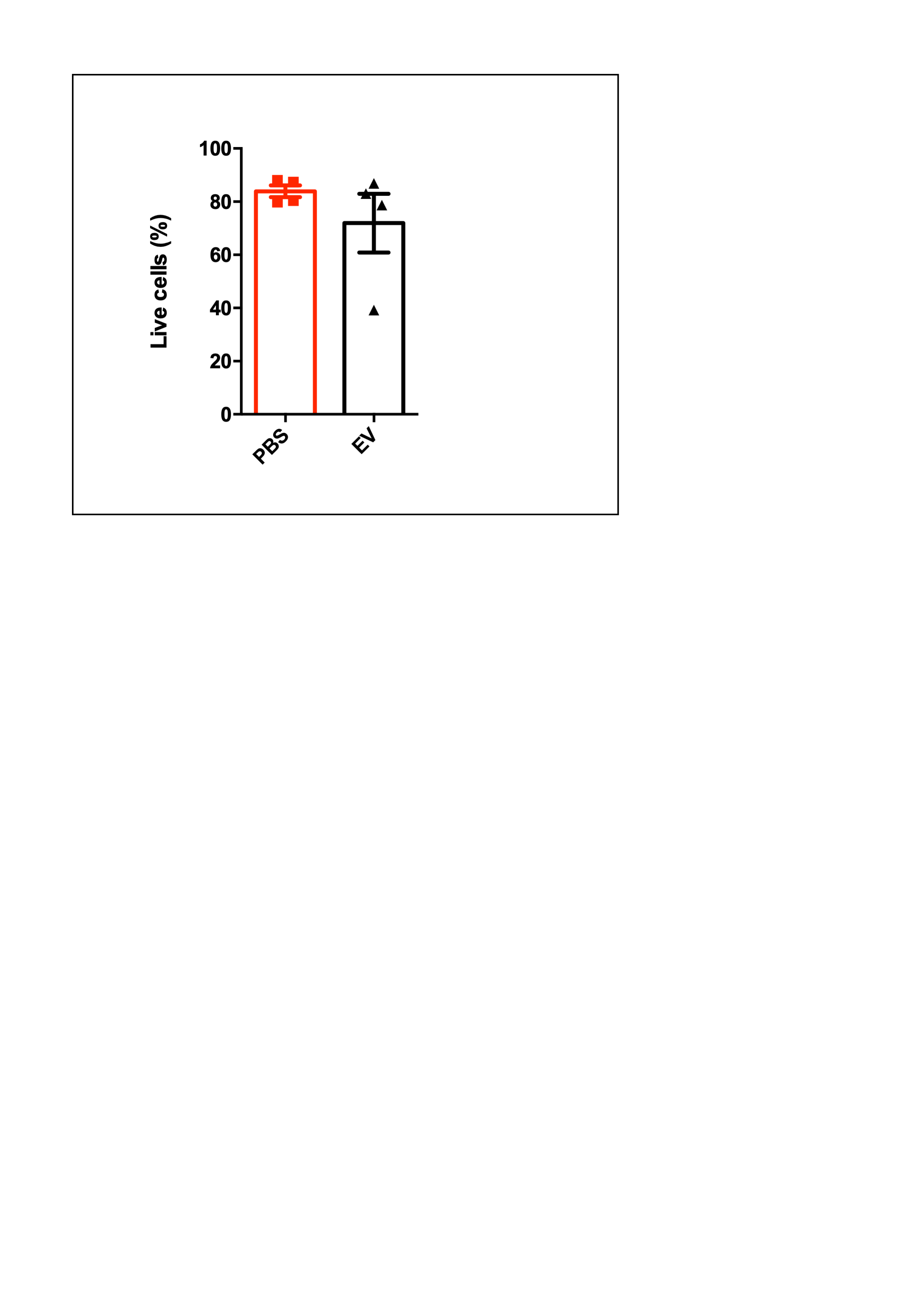

Supplement: Supplementary file 1 [file cells-09-01059-s001.zip › FigS1.tiff]

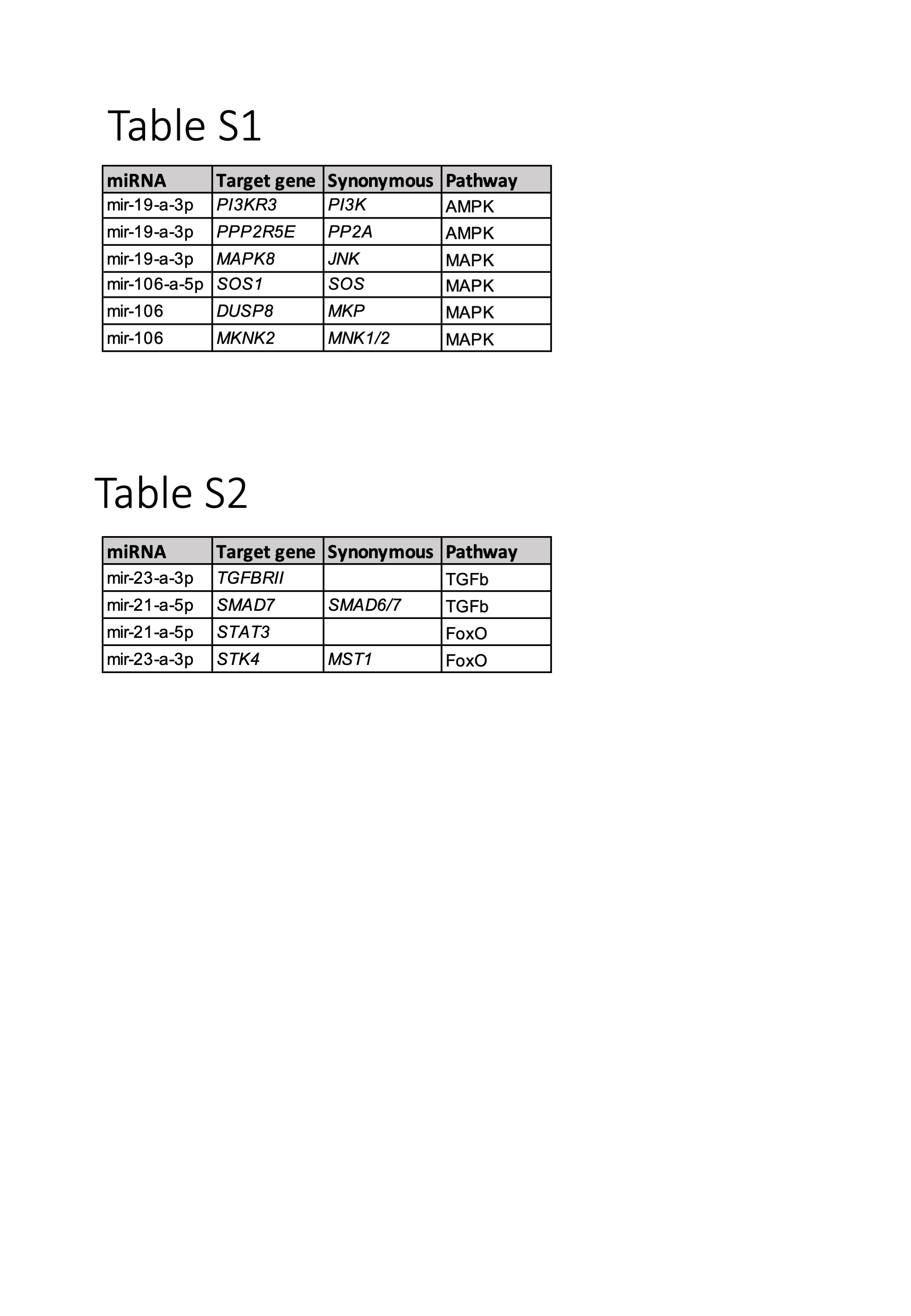

Supplement: Supplementary file 1 [file cells-09-01059-s001.zip › Tables.tiff]
